# Therapeutic potential of metabotropic glutamate receptor 7 modulation in correcting cognitive deficits of schizophrenia

**DOI:** 10.1016/j.schres.2026.06.012

**Published:** 2026-06-17

**Authors:** Niki M. Harris, Kimberly M. Holter, Colleen M. Niswender

**Affiliations:** a Vanderbilt Brain Institute, United States; b Department of Pharmacology and Warren Center for Neuroscience Drug Discovery, United States; c Vanderbilt Institute of Chemical Biology, Vanderbilt University, Nashville, TN, 37232, United States; d Vanderbilt Kennedy Center, Vanderbilt University Medical Center, Nashville, TN 37232, United States

**Keywords:** Metabotropic glutamate receptor 7, NMDA receptors, Therapeutics, Schizophrenia, Cognition, Prefrontal cortex

## Abstract

Cognitive deficits in schizophrenia are a major predictor of patient functional outcome, and current treatments have limited efficacy in targeting this symptom domain. Glutamatergic dysfunction is hypothesized to contribute to cognitive impairments, and administration of *N*-methyl-D-aspartate (NMDA) receptor antagonists to both healthy humans and animals causes behavioral and cognitive deficits similar to those observed in patients with schizophrenia. In particular, hypofunction of NMDA receptors on GABAergic interneurons in the medial prefrontal cortex (mPFC) leads to excessive pyramidal cell activity and is a hypothesized mechanism underlying these deficits. As such, therapeutic modulation of glutamatergic and GABAergic release in PFC-associated circuits may correct cognitive impairments in schizophrenia. Metabotropic glutamate (mGlu) receptors have emerged as a promising strategy to target this symptom domain due to their widespread expression in relevant brain regions and their implications within the glutamate system. Group III mGlu receptors (mGlu_4/6/7/8_) are G_i/o_-coupled receptors which predominantly show presynaptic localization and are well-positioned to modulate both glutamatergic and GABAergic neurotransmission. In particular, mGlu_7_ is widely expressed throughout the central nervous system, including expression on neurons within relevant PFC circuits. Interestingly, schizophrenia and other neuropsychiatric disorders characterized by cognitive deficits are associated with loss-of-function variants in *GRM7,* the gene encoding mGlu_7_. Furthermore, both mGlu_7_ potentiation and inhibition have shown efficacy in preclinical models of neurodevelopmental and neuropsychiatric disorders characterized by cognitive impairments. Therefore, mGlu_7_ is proposed to be a viable therapeutic target to treat cognitive deficits driven by glutamatergic dysfunction in disorders including schizophrenia.

## 1. Metabotropic glutamate (mGlu) receptors in disorders associated with cognitive impairments

### 1.1. Biology of mGlu receptors

Metabotropic glutamate (mGlu) receptors are class C G-protein coupled receptors that modulate glutamatergic and GABAergic neurotransmission in the central nervous system (Niswender and Conn, 2010). They are divided into three groups characterized by G-protein coupling profiles, pharmacological selectivity, intracellular signaling pathways, and sequence homology (Niswender and Conn, 2010). Group III mGlu receptors consist of subtypes mGlu_4_, mGlu_6,_ mGlu_7_, and mGlu_8_. The three widely expressed group III mGlu receptors (mGlu_4_, _7_, _8_), particularly mGlu_7_, are primarily localized to the active zone of pre-synaptic terminals, where they modulate glutamate or GABA release via inhibitory G_i/o_ coupling (Dalezios et al., 2002; Kinoshita et al., 1998; Shigemoto et al., 1996; Corti et al., 2002). Activation of group III mGlu receptors reduces intracellular cyclic AMP (cAMP) levels and protein kinase A (PKA) activity, inhibits voltage-gated calcium channels, and may disrupt synaptic vesicle priming (Niswender and Conn, 2010; Cartmell and Schoepp, 2000; Martín et al., 2010, 2007). Group III mGlu receptors, therefore, play an important role in modulation of synaptic transmission.

### 1.2. Group III mGlu receptor association with cognition

Dysregulation of group III mGlu receptors has been observed in several neuropsychiatric disorders, including schizophrenia. In human patients, polymorphisms in *GRM7* and *GRM4* – the genes encoding for mGlu_7_ and mGlu_4_, respectively – are associated with disorders characterized by cognitive deficits, such as attention-deficit/hyperactivity disorder (Zhang et al., 2021; Park et al., 2013), major depressive disorder (Niu et al., 2017; Li et al., 2016a; Gray et al., 2015), autism spectrum disorder (Noroozi et al., 2016), and schizophrenia (Li et al., 2016a; Zhang et al., 2018; Azari et al., 2019; Chaumette et al., 2022). mGlu_7_ is the most widely expressed metabotropic glutamate receptor in the central nervous system (Kinoshita et al., 1998; Ohishi et al., 1995; Kinzie et al., 1995; Saugstad et al., 1994; Shigemoto et al., 1996) and is expressed in brain regions involved in cognitive processes including the prefrontal cortex (PFC), striatum, hippocampus and thalamus (Ngomba et al., 2011). *GRM7* may be a risk gene for neurodevelopmental disorders as several mutations have been identified (Freitas and Niswender, 2023). Recent clinical studies evaluating 16 separate patients have revealed that biallelic mutations in *GRM7* are associated with developmental delay, intellectual disability, and epilepsy (Marafi et al., 2020; Jdila et al., 2021; Januel et al., 2024; Fisher et al., 2021; Freitas and Niswender, 2023).

In vivo, global mGlu_7_ knockout (KO) mice demonstrate deficits in associative fear learning, short-term synaptic plasticity, social behavior, motor coordination and strength; additionally, these mice exhibit handling-induced seizures (Fisher et al., 2020; Masugi et al., 1999; Sansig et al., 2001; Bushell et al., 2002), supporting the involvement of mGlu_7_ in normal behavioral and cognitive functioning. mGlu_7_ KO mice also show a significant reduction in hippocampal mRNA encoding the major isoforms of the proteins responsible for synthesizing GABA, GAD67 and GAD65 (Wierońska et al., 2010). Activation of mGlu_7_ at hippocampal GABAergic synapses leads to depression of GABAergic neurotransmission – a process proposed to be permissive for memory formation and induction of long-term potentiation (LTP) (Klar et al., 2015). Furthermore, mGlu_7_ potentiation reverses cognitive impairments within a mouse model of Rett syndrome – a disorder characterized by significant cognitive deficits – by correcting deficits in novel object recognition, contextual fear freezing behavior, apneas, and social behavior (Gogliotti et al., 2017). Collectively, these data are consistent with the hypothesis that mGlu_7_ plays a particularly important role in cognition.

### 1.3. Metabotropic glutamate receptor 7 (mGlu_7_) in schizophrenia

Within the context of schizophrenia, evaluation of 43 single nucleotide polymorphisms (SNPs) across all groups of mGlu receptors found that one SNP in *GRM7* – rs1396409 – was significantly associated with a lower performance IQ in a population of patients with first-episode psychosis, suggesting that mGlu_7_ variants may be associated with deficits in cognition during the early onset of the disorder (Chaumette et al., 2022). Furthermore, a meta-analysis across three genome-wide association studies (GWAS) identified an additional *GRM7* variant associated with schizophrenia diagnosis, rs1516569. This study also demonstrated that multiple intronic variants of *GRM7* predicted patient response to 7 common antipsychotic medications (Liang et al., 2020). Variants in *GRM7* may therefore not only be a predictor of risk of schizophrenia onset and associated cognitive deficits, but also of treatment response. Collectively, these data highlight a role for mGlu_7_ in schizophrenia symptoms and the receptor presents as a potentially promising therapeutic target to mitigate cognitive deficits.

## 2. Cognitive deficits in schizophrenia

Schizophrenia is a debilitating disorder characterized by positive, negative, and cognitive symptoms (Andreasen et al., 1995) affecting approximately 1% of the global population (Solmi et al., 2023). An estimated 70–90% of patients with schizophrenia worldwide exhibit cognitive impairments (Goonathilake et al., 2022; Arunpongpaisal and Sangsirilak, 2013; Kremen et al., 2000; Reichenberg et al., 2006; Fioravanti et al., 2012). According to the Measurement and Treatment Research to Improve Cognition in Schizophrenia (MATRICs) initiative, these impairments are characterized under seven primary domains which include working memory, speed of processing, attention, reasoning and problem solving, verbal and visual learning, and social cognition (Nuechterlein et al., 2008; Kern et al., 2008; Nuechterlein et al., 2023). Importantly, cognitive symptoms are highly correlated with functional outcomes in society and represent a major unmet need in the current therapeutic landscape. Impairments in social relationships, occupational integration, and ability to complete daily activities costs the United States an estimated $350 billion annually; unemployment and caregiving costs represent two of the largest contributing factors (Foussias et al., 2014; Ho et al., 1998; Green et al., 2000; Green, 1996; Horan et al., 2023; Kadakia et al., 2022; Green et al., 2019). Current antipsychotic medications primarily target serotonin and dopamine pathways and are effective against positive symptoms, which include hallucinations and delusions. Historically, the primary mechanism of action for antipsychotics was D_2_ receptor antagonism, with the addition of serotonergic targets in second-generation antipsychotics (Leucht et al., 2024). However, both first- and second-generation antipsychotics are often accompanied by adverse effects and do not effectively treat cognitive deficits (McCutcheon et al., 2023). Thus, it is imperative to identify therapeutics that effectively target all symptom clusters of the disease, including cognitive symptoms.

### 2.1. Cognitive deficits as an early predictor of schizophrenia

While most studies investigating cognitive deficits in schizophrenia focus on patients who have already been diagnosed, premorbid cognitive disruptions as early as childhood may serve as a predictor for future disease onset. For example, Davidson et al. matched the Israeli Draft Board Registry with the National Psychiatric Hospitalization Case Registry to identify relationships between early behavioral and intellectual assessments and later diagnosis with schizophrenia (Davidson et al., 1999). This study found that male adolescents who were later hospitalized and diagnosed with schizophrenia exhibited deficits in social, organizational, and intellectual abilities during adolescent years, suggesting that early deficits in these categories are a strong indicator for development of schizophrenia later in life (Davidson et al., 1999). Furthermore, an additional study using standardized cognitive test scores from kindergarten through grade 12 investigated the prevalence of cognitive deficits developed prior to schizophrenia diagnosis. The Iowa Tests of Basic Skills and the Iowa Tests of Educational Development scores were used for this study, both of which collectively evaluate skills such as reading comprehension, language, mathematics, sources of information, and vocabulary. Results showed that children who later developed schizophrenia exhibited declining scores between grades 8 and 11 (between the ages of 13 and 16), falling significantly below the state average by grade 11, and exhibiting a significant linear decrease in language scores across time (Fuller et al., 2002). Thus, cognitive deficits are not only a highly prevalent symptom associated with schizophrenia, but early signs of cognitive impairment may also serve as a predictor for development of schizophrenia later in life.

## 3. The role of cortical circuit dysfunction in cognitive symptoms of schizophrenia

Dysfunction in the PFC has been implicated in underlying cognitive impairments in schizophrenia. In support, functional connectivity data from patients with schizophrenia highlight disruptions in the PFC (Baker et al., 2014), specifically in regions known to contribute to cognitive function, such as working memory, attention, and executive function (Sugranyes et al., 2012; He et al., 2020). Early-stage patients with schizophrenia exhibit PFC hyperfunctional connectivity (Anticevic et al., 2015) and, in patients with first-episode psychosis, increased PFC glutamate concentrations have been observed (Natsubori et al., 2014). Within the PFC, evidence shows dysfunctional connectivity between dorsal and ventral medial PFC (Ćurčić-Blake et al., 2015), later-alized dorsolateral PFC engagement (Huang et al., 2024), and decreased activity of the anterior PFC (Kaplan et al., 2016), highlighting the relationship between prefrontal cortical disruptions and schizophrenia.

Many circuits involving the PFC show additional connectivity abnormalities. For example, patients exhibit decreased connectivity between PFC-somatomotor and default mode networks (He et al., 2020). Further, patients display reduced prefrontal-thalamic connectivity (Woodward et al., 2012) as well as hypoconnectivity of cortico-striatal salience networks (Karcher et al., 2019) which are specifically linked to cognitive impairment (Avram et al., 2018). Hippocampal-cortical connections have long been studied to elucidate their role in cognition (Li et al., 2015; Sigurdsson and Duvarci, 2016). Abnormal PFC-hippocampus connectivity has been observed in patients with schizophrenia (Sigurdsson and Duvarci, 2016) and is associated with cognitive deficits such as impairment in associative memory (Zhai et al., 2025). Collectively, these functional data in clinical populations support the strong role of cortical circuit dysfunction in cognitive deficits associated with schizophrenia.

## 4. Evidence for NMDA receptor hypofunction in cognitive deficits of schizophrenia

### 4.1. NMDA receptor function

A growing body of literature suggests that glutamatergic dysfunction underlies cognitive impairments in schizophrenia, with strong evidence for hypofunction of *N*-methyl-D-aspartate (NMDA) receptors. NMDA receptors are heteromeric ionotropic glutamate receptors, containing two GluN1 obligatory subunits and either two GluN2 subunits and/or two GluN3 subunits (Hansen et al., 2021; Zhou and Tajima, 2023; Dupuis et al., 2023). These receptors require binding of both glutamate to the GluN2 subunit and either glycine or d-serine to the GluN1 subunit, respectively (Zhou and Tajima, 2023; Yu et al., 2018; Hansen et al., 2021). At rest, channel activity is inhibited by voltage-dependent Mg^2+^ blockade (Mayer et al., 1984), which can be released by depolarization of the membrane via activation of α-amino-3-hydroxy-5-methyl-4-isoxazolepropionic acid (AMPA) receptors. Mg^2+^ blockade, therefore, acts as a coincidence detection mechanism, and these receptors require both membrane depolarization and presynaptic glutamate release to allow for an influx of positive ions (Hansen et al., 2021). Due to these properties, NMDA receptors and their specific subunit composition play a pivotal role in synaptic signaling and influence both learning and memory (Paoletti et al., 2013). Furthermore, NMDA receptor subunit composition shifts throughout development. GluN2B subunit expression levels decrease with age, while GluN2A expression levels increase, becoming the predominant subunit in late childhood and adolescence (Singh et al., 2022; Monyer et al., 1994; Dupuis et al., 2023). The shift in the predominantly expressed NMDA receptor subunits plays a key role in neuronal maturation and plasticity (Dupuis et al., 2023); therefore, any abnormalities may have vast implications on cognitive processes.

### 4.2. NMDA receptors and mGlu_7_

Additionally, NMDA receptors may interact with mGlu_7_, contributing to the modulation of NMDA receptor activity. While mGlu_7_ is predominantly expressed in presynaptic terminals, electron microscopy studies have revealed some additional postsynaptic expression (Kosinski et al., 1999; Brandstätter et al., 1996), suggesting that mGlu_7_ may be positioned to modulate NMDA receptor activity via a postsynaptic mechanism (Bradley et al., 2012; Gu et al., 2012). Application of AMN082, an agonist with activity at mGlu_7_, to hippocampal primary cultured neurons induces a significant increase in phosphorylated GluN1 and GluN2B protein levels, but not a significant change in total protein expression (Bradley et al., 2012). In contrast, an additional study found that application of the group III mGlu receptor agonist L-2-Amino-4-phosphonobutyrate (L-AP4) decreases the density of NMDA receptor membrane surface expression; this decrease in surface expression may be mediated by mGlu_7_ interference with NMDA receptor trafficking (Gu et al., 2012). In addition to protein expression, mGlu_7_ activity may modulate neuronal currents driven by NMDA receptor activity. For example, following application of L-AP4, NMDA receptor-mediated currents were reduced in a region with high expression of mGlu_7_ and no expression of other group III receptors; this effect is blocked by group III mGlu receptor antagonists (Gu et al., 2014). Bath application of L-AP4 on PFC pyramidal neurons reduces NMDA receptor-mediated excitatory postsynaptic currents (EPSCs); this effect is lost following siRNA-mediated mGlu_7_ knockdown, suggesting that mGlu_7_ activity suppresses NMDA receptor currents via a postsynaptic mechanism (Gu et al., 2012). These studies suggest that mGlu_7_ may play a role in NMDA receptor function, further supporting the role of mGlu_7_ in dysregulated glutamatergic signaling.

### 4.3. Evidence of NMDA receptor hypofunction in schizophrenia

Disruptions in NMDA receptor function may drive abnormal glutamatergic signaling and contribute to schizophrenia symptomology. In support of this hypothesis, one study using data from patients with schizophrenia or autism spectrum disorder found 38 missense mutations, one frameshift mutation, and one splice site mutation in the genes encoding NMDA receptor subunits including *GRIN1, GRIN2A, GRIN2C, GRIN2D, GRIN3A*, and *GRIN3B* (Yu et al., 2018), suggesting that dysfunctional NMDA receptors may be associated with disorders like schizophrenia. Moreover, animals with a genetic knockout of GluN1 in either pyramidal cells or GABAergic interneurons exhibit cognitive and behavioral deficits similar to those seen in patients with schizophrenia (Nakazawa et al., 2017). Mice with genetic knockout of GluN2B subunits, specifically in pyramidal cells in the forebrain, also exhibit hyperlocomotion – a correlate for positive symptoms – as well as learning and memory impairments (von Engelhardt et al., 2008; Badanich et al., 2011). Furthermore, mice with a genetic knockout of GluN2A subunits demonstrate a hyperactive phenotype, show reduced nest building, impaired social novelty preference, and a spatial working memory deficit, suggesting that loss of GluN2A recapitulates a schizophrenia-like phenotype (Lu et al., 2024). Therefore, NMDA receptor subtypes differentially contribute to cognitive functioning, and subtype-specific deficits may underlie symptoms of schizophrenia.

Additional evidence pointing to NMDA receptor hypofunction stems from patients with the autoimmune disorder anti-NMDA receptor encephalitis, which results from autoantibodies that primarily target GluN1 subunits, leading to receptor internalization (Gleichman et al., 2012). These patients exhibit symptoms resembling those seen in schizophrenia, including delusions, hallucinations, and catatonia (Subeh et al., 2021), supporting the link between NMDA receptor hypofunction and psychosis. Hippocampal neurons incubated with NMDA receptor-Ab IgG from patients with schizophrenia show altered surface trafficking of NMDA receptors and impaired NMDA receptor-dependent LTP (Jézéquel et al., 2017). Studies have since utilized NMDA receptor-IgG from patients with anti-NMDA receptor encephalitis to induce cognitive and behavioral deficits in rodents; these animals exhibit decreased sucrose consumption, increased immobility in a forced swim test, and decreased prepulse inhibition (Villéga et al., 2024).

Lastly, there is pharmacological evidence from early clinical studies showing that administration of NMDA receptor antagonists, such as phencyclidine (PCP) or ketamine, to healthy human subjects results in behavioral and cognitive abnormalities similar to those seen in patients with schizophrenia (Krystal et al., 1994; Newcomer et al., 1999; Lahti et al., 2001). Furthermore, administration of an NMDA receptor antagonist to patients with schizophrenia exacerbates preexisting symptoms (Lahti et al., 1995; Luby et al., 1959). Interestingly, patients with schizophrenia also display increased levels of kynurenic acid in cerebrospinal fluid (Linderholm et al., 2012) and blood (Hare et al., 2023), which serves as an antagonist of NMDA receptors (Kindler et al., 2020) and induces cognitive deficits (Pocivavsek et al., 2012; Hare et al., 2023). Additionally, rodent tasks can be utilized to evaluate these cognitive deficits, including evaluation of memory through the Morris water maze (Ning et al., 2017; Enomoto et al., 2008), Y maze (Mabunga et al., 2019), and novel object recognition (de Lima, 2005) tasks, as well as behavioral evaluation through a social preference task (Mabunga et al., 2019). Impairments across these tasks, including deficits in working memory (Driesen et al., 2013; Vinnakota et al., 2024; Holter et al., 2021), social behavior (Chaki et al., 2015), and attention (Holter et al., 2025; Patrono et al., 2023; Szlachta et al., 2017) can be recapitulated in animals following administration of NMDA receptor antagonists including PCP, ketamine, and MK-801. Therefore, NMDA receptor antagonists are valuable tools to induce a state of NMDA receptor hypofunction in rodents, which can be utilized to test efficacy of novel therapeutics against glutamatergic dysfunction and associated cognitive deficits.

## 5. The roles of GABAergic interneurons and pyramidal cells in NMDA receptor hypofunction

Evidence suggests that the medial PFC (mPFC) and its associated neuronal subpopulations, including pyramidal cells and GABAergic interneurons, may contribute to the cognitive impairments observed in schizophrenia (Vogel et al., 2022; Yang et al., 2021; Horst and Laubach, 2009; Abbas et al., 2018). Patients exhibit a decrease in number, soma volume, and density of mPFC pyramidal cells (Larsen et al., 2022), as well as a reduction in spine density (Kolluri et al., 2005). The role of the mPFC in cognitive function is supported in preclinical work via the disruption of mPFC circuitry in rodent models. For example, optogenetic silencing of mPFC pyramidal cells, as well as GABAergic interneurons, leads to impaired performance in a T-maze task, suggesting that both cellular populations contribute to spatial working memory (Vogel et al., 2022; Abbas et al., 2018). Taken together, these data support the hypothesis that maintaining stable cognitive performance requires a balance between excitatory pyramidal cells and inhibitory GABAergic interneurons; aberrant shifts in excitatory/inhibitory (E/I) balance underlie cognitive impairments for a variety of neuropsychiatric disorders including schizophrenia (Yizhar et al., 2011; Sohal and Rubenstein, 2019; Ferguson and Gao, 2018).

NMDA receptor hypofunction therefore contributes to an imbalance between activity of these excitatory pyramidal cells and inhibitory GABAergic interneurons. Acute systemic administration of the NMDA receptor antagonist MK-801 induces a state of NMDA receptor hypofunction, preferentially targeting GABAergic interneurons (Homayoun and Moghaddam, 2007). MK-801 administration reduces spontaneous inhibitory transmission onto mPFC glutamatergic pyramidal cells, leading to disinhibition, downstream increases in glutamate signaling and hyperexcitability, and is associated with observed cognitive deficits (Homayoun and Moghaddam, 2007; Luessen et al., 2022; Jackson et al., 2004; Conn et al., 2009). Interestingly, infusion of NMDA receptor antagonists locally into the mPFC does not increase pyramidal cell excitation, whereas infusion into the hippocampus does increase excitation (Jodo et al., 2005). These data suggest that MK-801-induced aberrant pyramidal cell signaling in the mPFC may be a result of reduced inhibitory inputs from regions outside of the mPFC. Collectively, evidence suggests that therapeutics that normalize cortical glutamatergic signaling may be optimal for targeting cognitive disruptions in schizophrenia.

Lines of evidence highlight a potential role of NMDA receptor hypofunction on parvalbumin-expressing interneurons (PV-INs) in schizophrenia. Clinically, patients exhibit a significant reduction in PV-INs that express mRNA for the GluN2A subunit, suggesting that hypofunction of GluN2A-containing NMDA receptors on PV-INs may contribute to symptomology (Bitanihirwe et al., 2009). Wider support stems from preclinical models in which MK-801 administration decreases PV-IN density in the mPFC (Li et al., 2016c). While administration of MK-801 inhibits rodent performance in an attentional set-shifting task, optogenetic stimulation of PV-INs within the PFC reverses these cognitive impairments (Patrono et al., 2023). Additional studies show that both low and high doses of MK-801 chronically administered for 5 consecutive days can significantly affect NMDA receptor subunit expression in PV-INs via decreases in mRNA expression of *GRIN1* and *GRIN2A* (Xi et al., 2009). Therefore, preclinical and clinical evidence for hypofunction of NMDA receptors on PV-INs suggest PV-INs may strongly contribute to cognitive impairments in patients with schizophrenia.

In addition to PV-INs, abnormalities in somatostatin-expressing interneurons (SST-INs) are also implicated in schizophrenia and hypothesized to contribute to cognitive deficits. SST-INs target apical dendrites of pyramidal cells as well as other interneurons, including PV-INs, to regulate their inhibitory action onto pyramidal cells (Abbas et al., 2018; Pfeffer et al., 2013; Van Derveer et al., 2020). Somatostatin release is mediated by activation of NMDA and AMPA receptors, suggesting that NMDA receptor dysfunction may have aberrant effects on SST-INs (Song et al., 2021; Tapia-Arancibia and Astier, 1989; Fontana et al., 1996). Preclinical evidence suggests that SST-INs are necessary for cognitive flexibility, social behavior, and memory consolidation in fear conditioning (Cummings and Clem, 2020; Perez et al., 2019). Enhanced SST-IN activity contributes to the correction of MK-801-induced working memory deficits – an effect that does not occur when SST-INs are optogenetically inhibited – providing further evidence that SST-INs are implicated in cognitive function (Maksymetz et al., 2021). Clinical evidence further demonstrates the role of SST-INs in cognitive function as patients with schizophrenia show decreased expression of SST mRNA in the PFC (Dienel et al., 2023; Morris et al., 2008). These data highlight the relevance of PFC SST-IN signaling for multiple cognitive domains, suggesting that targeting these synapses may ameliorate deficits in patients with schizophrenia.

## 6. Current pharmacological approaches to ameliorate NMDA receptor hypofunction & their limitations

### 6.1. Historical dopaminergic approaches to pharmacology

Current FDA-approved treatments for schizophrenia attenuate positive symptoms, while cognitive symptoms remain largely untreated (Keefe et al., 2007; Meltzer, 1999; McCutcheon et al., 2023). These medications include antipsychotic drugs categorized as first-generation (typical) and second-generation (atypical) antipsychotics. Both first- and second-generation antipsychotics are associated with a large adverse effect profile, including extrapyramidal and metabolic symptoms (Seeman, 2002; Li et al., 2016b; Stępnicki et al., 2018; Orzelska-Górka et al., 2022; Horacek et al., 2006; Lally and MacCabe, 2015; Meltzer, 2013; Carli et al., 2021). Importantly, these first and second-generation antipsychotics show little efficacy in treating cognitive symptoms (Feber et al., 2025). A newer “third” generation of antipsychotics, including brexpiprazole and aripriprazole, exhibit more promising safety and tolerability profiles (Newcomer et al., 2018; Watanabe et al., 2020; Vasiliu, 2022), though efficacy against cognitive symptoms is still limited (Orzelska-Górka et al., 2022; Stępnicki et al., 2018). Thus, targeting dopaminergic or serotoninergic pathways may not sufficiently mitigate the cortical disruptions implicated in schizophrenia-associated cognitive impairments.

### 6.2. Novel alternative pharmacological approaches

Alternative approaches aiming to normalize glutamatergic signaling have been explored as a potential therapeutic strategy for schizophrenia through both the direct and indirect modulation of NMDA receptors. As memantine, a low-affinity NMDA receptor antagonist, gained traction as a pro-cognitive drug for Alzheimer’s disease (Karimi Tari and Parsons, 2024; Swerdlow et al., 2016), it was also explored in schizophrenia, showing beneficial effects on disrupted sensorimotor gating and negative symptoms in the clinic (Swerdlow et al., 2016; Zheng et al., 2019). However, results are variable as other studies show that memantine does not significantly improve MATRICS Consensus Cognitive Battery performance (Bhakta et al., 2016). As such, additional mechanisms involving NMDA receptor modulation have been explored. Glycine modulation, both directly and indirectly, has shown promise for the treatment of all symptom clusters of schizophrenia, as directly targeting the glycine modulatory site in NMDA receptors can reverse cognitive and social deficits induced by PCP in preclinical models (Pei et al., 2021; Mouri et al., 2007). Clinically, administration of glycine has shown variability in improvement of cognitive symptoms of patients (Buchanan et al., 2007; Evins et al., 2000; Heresco-Levy et al., 2004). Further, the NMDA receptor modulator d-serine has shown efficacy for the treatment of negative symptoms (Kantrowitz et al., 2026) and improves performance in auditory mismatch negativity – a neurophysiological sensory domain in which impairments are correlated with poor functional outcome for patients (Kantrowitz et al., 2018; Sehatpour et al., 2023). However, clinical studies have thus far shown no significant difference in cognition as measured by the MATRICS battery (Zimmerman et al., 2012; Kantrowitz et al., 2018). Therefore, D-amino acid oxidase inhibitors (DAOIs), which indirectly modulate d-serine to hinder its metabolism were investigated. A recent meta-analysis examining clinical trials for these compounds reveals promise in improving cognitive function; however, due to the small sample size of trials, future studies are needed to confirm these findings (Chang et al., 2025). Further, a phase 2 trial of the selective DAOI luvadaxistat was recently completed and did not show a significant difference between treatment vs placebo in the Brief Assessment of Cognition in Schizophrenia composite score (Khin et al., 2026). Additional meta-analyses examining NMDA receptor modulators, including co-agonists and DAOIs, reveal that while receptor modulation may be efficacious against positive and negative symptoms (Goh et al., 2021), it may not effectively treat cognitive symptoms (Chang et al., 2019), suggesting that alternative mechanisms of action may better ameliorate this symptom domain.

Indirect modulation of glycine has thus alternatively been explored via inhibition of the glycine transporter type 1 (GlyT1) to regulate extracellular glycine levels and correct dysfunctional glutamatergic signaling (Zafra et al., 2017; Singer and Yee, 2024). In rodent models of mutant GlyT1, NMDA receptor EPSC amplitude is significantly increased, and rodents show an improvement in associative learning, suggesting that disruption of GlyT1 function may exert pro-cognitive effects (Yee et al., 2006; Gabernet et al., 2004). GlyT1 inhibitors, such as Bitopertin and Sarcosine, were developed and demonstrated promising findings for cognitive and behavioral abnormalities in preclinical studies (Black et al., 2009; Rosenbrock et al., 2022; Chen-Engerer et al., 2022; Kumar et al., 2023; Deiana et al., 2022), leading to advancement of GlyT1 inhibitors into clinical trials for treatment of cognitive deficits in schizophrenia (Rosenbrock et al., 2023; Harvey et al., 2025). However, many clinical trials involving GlyT1 inhibitors have been halted (Cioffi, 2018; Coyle and Paul, 2026) due to either adverse effects or lack of efficacy. Others, such as Iclepertin (BI 425809), did not meet designated primary and secondary endpoints in Phase III trials, leading to the discontinuation of long-term trials (Boehringer Ingelheim, 2025).

Cobenfy^®^ (Xanomeline-Tropsium Chloride), a drug recently approved by the US Food and Drug Administration, represents an alternative approach, serving as a nonselective muscarinic agonist with the strongest activity at muscarinic acetylcholine M_1_ and M_4_ receptors (Zheng et al., 2018; Commissioner, 2024; Paul et al., 2022). Post-synaptic G_q_-coupled M_1_ receptors on GABAergic interneurons increase inhibition onto both dopamine neurons and pyramidal cells, thereby attenuating glutamatergic signaling in the PFC (Meyer et al., 2025). Interneuron activation, coupled with the potentiation of presynaptic G_i/o_-coupled M_4_ receptors, further diminishes dopamine release in the ventral tegmental area, nucleus accumbens, and striatum (Smith et al., 2025). In clinical trials, Cobenfy administration shows improvements in both positive and negative symptoms (Meyer et al., 2025). Further, patients with more severe cognitive impairments at baseline who received Cobenfy exhibited improvements in cognition compared to placebo, though this effect was not maintained in pooled data across trials (Smith et al., 2025; Horan et al., 2025; Hasan et al., 2024). Therefore, Cobenfy trials have provided foundational evidence for modulation of glutamatergic, dopaminergic, and cholinergic systems as a therapeutic strategy for cognitive deficits in schizophrenia. However, despite this substantial progress in the treatment landscape for schizophrenia, there remains a need to develop more selective treatment options that address cognitive impairments across the full patient population.

### 6.3. Modulation of group I and II mGlu receptors

An additional strategy that has been explored for treatment in schizophrenia is modulation of the glutamatergic pathway via group I and group II mGlu receptor (mGlu_1/5_ and mGlu_2/3_, respectively) potentiation (Olivares-Berjaga et al., 2025; Luessen and Conn, 2022). Group I mGlu receptors have shown extensive preclinical promise in the treatment of schizophrenia (Nicoletti et al., 2019; Luessen and Conn, 2022). mGlu_5_ interacts with NMDA receptors and may potentiate NMDA receptor-mediated currents, suggesting that activation of this receptor may normalize excitatory/inhibitory imbalance (Awad et al., 2000; Collett and Collingridge, 2004). Excitingly, potentiation of both mGlu_1_ and mGlu_5_ demonstrates preclinical efficacy in correcting cognitive and behavioral abnormalities associated with schizophrenia-like pathophysiology (Maksymetz et al., 2021; Nicoletti et al., 2019; Horio et al., 2013; Stefani and Moghaddam, 2010; Brown et al., 2022). However, although the antipsychotic effects are promising, the reported adverse effects following administration of these compounds has limited their clinical translation (Parmentier-Batteur et al., 2014; Rook et al., 2015; Reed et al., 2024; Sturm et al., 2018). Thus, while mGlu_5_ PAMs have entered clinical trials, studies have been discontinued due to adverse events (Sturm et al., 2018).

Strong preclinical evidence also suggests that modulation of group II receptors exhibits efficacy across all symptom domains, including cognition (Nikiforuk et al., 2010; Cieślik et al., 2019; Lavreysen et al., 2015; Martínez-Pinteño et al., 2023). As such, both the mGlu_2/3_ agonist LY2140023 and the mGlu_2_ positive allosteric modulator (PAM) AZD8529 entered clinical trials to evaluate their efficacy in schizophrenia. However, in a phase II clinical study, neither AZD8529 nor LY2140023 demonstrated separation in efficacy from placebo (Kinon et al., 2011; Litman et al., 2016). Further, AZD8529 administration did not show improvement in a working memory task, suggesting that mGlu_2_ activity may not be sufficient to restore cognitive function in patients (Wolf et al., 2022). While these broader clinical trials may not have shown efficacy, ongoing work suggests that modulation of group II mGlu receptors may be efficacious in a smaller subgroup of early-in-disease patients, suggesting that a personalized approach may be necessary to show treatment efficacy (Kinon et al., 2015).

Overall, extensive preclinical and clinical work has been performed to investigate a variety of mechanisms to improve patient outcomes across symptom domains. However, many of these attempted mechanisms of action are either associated with adverse side effects or do not demonstrate significant differences in patient outcomes compared to placebo or currently available therapeutics in the treatment of cognitive deficits. Thus, there remains a need for development of new therapies for schizophrenia that target cognitive symptoms.

## 7. Therapeutic potential of targeting mGlu_7_

Activation of group III mGlu receptors, including mGlu_7_, is a novel potential therapeutic strategy for all symptom domains of schizophrenia, including cognitive impairments. In preclinical models of NMDA receptor hypofunction, administration of group III mGlu receptor agonists significantly reduces hyperlocomotive effects (Pałucha-Poniewiera et al., 2008; Wierońska et al., 2012). Among these group III mGlu receptors, mGlu_4_ potentiation in models of schizophrenia-like pathophysiology has shown efficacy in the correction of behavioral abnormalities such as hyperlocomotion, social withdrawal, and working memory deficits (Sławińska et al., 2013). However, observed procognitive effects following mGlu_4_ potentiation were blocked by the administration of a 5-HT_1A_ receptor antagonist, suggesting that mGlu_4_ alone may not be sufficient to ameliorate cognitive deficits (Wierońska et al., 2015). Interestingly, reduced levels of cinnabaric acid, an mGlu_4_-activating kynurenine metabolite, have been found in human patients with schizophrenia (Ulivieri et al., 2020). In preclinical studies, systemic cinnabaric acid corrects MK-801-induced hyperactivity and social behavioral deficits – an effect that is not observed in mGlu_4_ knockout mice (Ulivieri et al., 2020). Therefore, mGlu_4_ activation alone may be beneficial for the positive and negative symptoms of schizophrenia; however, effects on cognition remain unclear.

Of the group III mGlu receptors, mGlu_7_ shows particular therapeutic promise as it is widely expressed within regions affected in schizophrenia, including the hippocampus and cortex (Makoff et al., 1996). Group III mGlu receptor activation through the application of L-AP4 at concentrations that activate mGlu_7_ induces a decrease in hippocampal EPSC amplitude, suggesting that activation of these receptor subtypes depresses excitatory neurotransmission (Baskys and Malenka, 1991; Ayala et al., 2008). Further, mGlu_7_ exhibits presynaptic expression within PFC pyramidal cells (Stachniak et al., 2019; Ferraguti and Shigemoto, 2006; Reyes et al., 1998) – a promising position to normalize glutamate release. Recordings from PFC SSTs receiving input from pyramidal cells show a decrease in EPSCs when mGlu_7_ is activated via L-AP4 application (Lei et al., 2025), suggesting that mGlu_7_ activation suppresses excitatory synaptic signaling. Additionally, application of methylserine-O-phosphate (MSOP) or the negative allosteric modulator (NAM) ADX71743 to antagonize mGlu_7_ activity leads to increased EPSC amplitudes (Stachniak et al., 2019; Ciobanu et al., 2026). Together, these data suggest that potentiation of mGlu_7_ reduces excitatory PFC signaling. Therefore, in a state of NMDA receptor hypofunction and disinhibition of PFC pyramidal cells, mGlu_7_ may function to depress overactive glutamatergic transmission (Fig. 1). While this is one such proposed model, targeting mGlu_7_ to ameliorate schizophrenia symptoms has been explored in other ways. For example, NAMs of mGlu_7_ administered in preclinical models of schizophrenia have additionally demonstrated efficacy across symptom domains (Cieślik and Wierońska, 2022; Cieślik et al., 2018). This alternative model proposes that, due to expression of mGlu_7_ at GABAergic synapses, a reduction in mGlu_7_ activity may increase GABAergic release, effectively restoring excitatory/inhibitory balance and further supporting mGlu_7_ as a novel therapeutic target (Fig. 1) (Cieślik and Wierońska, 2022). While these models hypothesize opposing mechanisms of action, they share the same end goal of targeting mGlu_7_ to normalize excitatory/inhibitory imbalance (Fig. 1). Investigation into the exact mechanism of action by which mGlu_7_ exerts its therapeutic effects is still in progress; however, these compelling clinical and preclinical data suggest targeting mGlu_7_ is a promising strategy to potentially normalize the dysfunctional glutamatergic signaling that may underlie cognitive deficits in schizophrenia.

### 7.1. Limitations of mGlu_7_ modulation

While continued efforts emphasize the promising therapeutic effects of targeting mGlu_7_, mGlu_7_-specific ligands have historically been difficult to develop. Investigation into the activation of group III mGlu receptors led to the synthesis of PAMs with activity at mGlu_7_ (Jalan-Sakrikar et al., 2014). However, these compounds are not selective for mGlu_7_ and exhibit activity at other group III mGlu receptors. More recently, VU6046980 was developed as an mGlu_7_-selective PAM; however, off-target sedative effects were retained in *Grm7* KO mice (Kalbfleisch et al., 2023), suggesting that further work is needed for the development of mGlu_7_-selective ligands. Further, due to its wide central nervous system distribution (Ohishi et al., 1995; Saugstad et al., 1994) and postsynaptic interactions with NMDA receptors (Gu et al., 2012), it is unclear how difficult it will be to elicit selective effects in specific circuits. Despite these limitations, expansive bodies of both preclinical and human genetic data highlight the substantial need for novel therapeutic development targeting cognitive deficits and the potential of mGlu_7_ as one such target. As such, efforts should continue into the development of mGlu_7_-specific compounds.

## 8. Conclusions

There is a substantial need for therapeutics that target cognitive deficits in schizophrenia, which serve as a predictor for both development of psychosis and of patient functional outcomes. NMDA receptor hypofunction can lead to aberrant glutamatergic signaling, which is a potential pathological mechanism for cognitive symptoms. Further, dysfunctional neuronal activity and functional connectivity in the PFC are associated with deficits in several affected cognitive domains, suggesting that targeting relevant receptors in this circuitry may improve cognitive function. NMDA receptor antagonists impair cognitive performance in both humans and animals; evidence supports a greater impact of these antagonists on GABAergic interneurons within the mPFC, suggesting that downstream cortical hyperexcitability is driven by disinhibition of pyramidal cells. Therefore, therapeutics that normalize glutamatergic output may be a promising mechanism to target cognitive impairments in schizophrenia. mGlu_7_ is one possible target due to its ability to modulate glutamatergic and GABAergic signaling, thereby normalizing excitatory/inhibitory imbalance. While ongoing efforts continue to expand upon the therapeutic landscape for the treatment of schizophrenia, current therapeutics are limited in their efficacy against cognitive symptoms. Mechanistic insights into schizophrenia pathology point to mGlu_7_ as a promising therapeutic target, and, as drug discovery and development efforts continue to evolve, development of selective mGlu_7_ therapies may expand treatment options effective against all symptom domains.

## Figures and Tables

**Fig. 1.**
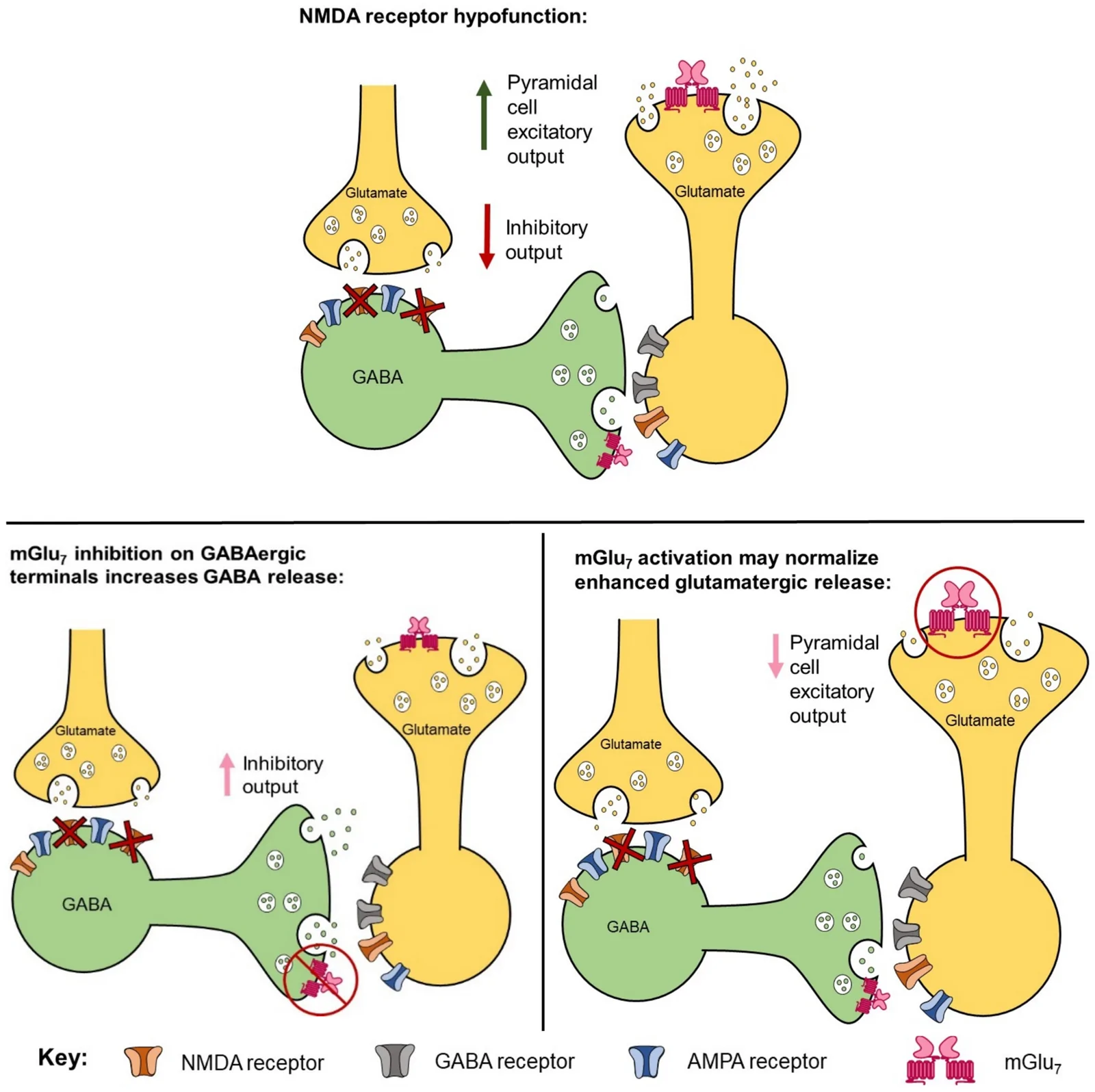
Presynaptic mechanisms for mGlu_7_ engagement in models of NMDA receptor hypofunction. NMDA receptor hypofunction on GABAergic interneurons induces enhanced release of glutamate via disinhibition onto pyramidal cells (top). mGlu_7_ (pink) acts as an autoreceptor and heteroreceptor to modulate glutamatergic and GABAergic release. Located predominantly on presynaptic terminals and coupled to G_i/o_ proteins, mGlu_7_ activation reduces cAMP levels and calcium channel signaling to regulate neurotransmission. In the context of NMDA receptor hypofunction, mGlu_7_ NAMs (left) may show efficacy by blocking regulatory mGlu_7_ action and increasing inhibitory output onto pyramidal cells. However, it should be noted that the activity of GABAergic interneurons is already blunted in models of NMDA receptor hypofunction, potentially minimizing the effect of mGlu_7_ modulation in these cell types. Alternatively, mGlu_7_ potentiation may reduce excessive glutamatergic transmission, effectively normalizing glutamatergic drive. Therefore, both proposed mechanisms may restore cognitive function in schizophrenia-like pathophysiology by normalizing excitatory/inhibitory imbalance. Lastly, postsynaptic expression has been reported (not shown), where mGlu_7_ has the potential to more directly regulate NMDA receptor function. (For interpretation of the references to colour in this figure legend, the reader is referred to the web version of this article.)
